# Mitochondrial Metabolism in Pancreatic Ductal Adenocarcinoma: From Mechanism-Based Perspectives to Therapy

**DOI:** 10.3390/cancers15041070

**Published:** 2023-02-08

**Authors:** Hafiza Padinharayil, Vikrant Rai, Alex George

**Affiliations:** 1 Jubilee Centre for Medical Research, Jubilee Mission Medical College and Research Institute, Thrissur 680005, Kerala, India; 2 Department of Translational Research, Western University of Health Sciences, Pomona, CA 91766-1854, USA

**Keywords:** pancreatic ductal adenocarcinoma, mitochondria, metabolism, retrograde signaling, extracellular matrix, immune cells, therapy

## Abstract

**Simple Summary:**

In cancer therapy, mitochondrial metabolism has emerged as a particularly attractive target, especially for malignancies such as pancreatic ductal adeno carcinoma (PDAC) that are resistant to treatment. Along with other treatment approaches, including targeting glutamine and fatty acid metabolism and inhibiting the tricarboxylic acid (TCA) cycle precursors, mitochondrial oxidative phosphorylation (OXPHOS) is still a key target in cancer therapy. To the advantage of PDAC patients, innovative and more effective therapeutics will be possible with a greater understanding of how pancreatic cancer cells control mitochondrial metabolism, and their role in PDAC progression through modulating cellular dynamics, bioenergetics, immune education, and retrograde signaling.

**Abstract:**

Pancreatic ductal adenocarcinoma (PDAC), the fourteenth most common malignancy, is a major contributor to cancer-related death with the utmost case fatality rate among all malignancies. Functional mitochondria, regardless of their complex ecosystem relative to normal cells, are essential in PDAC progression. Tumor cells’ potential to produce ATP as energy, despite retaining the redox potential optimum, and allocating materials for biosynthetic activities that are crucial for cell growth, survival, and proliferation, are assisted by mitochondria. The polyclonal tumor cells with different metabolic profiles may add to carcinogenesis through inter-metabolic coupling. Cancer cells frequently possess alterations in the mitochondrial genome, although they do not hinder metabolism; alternatively, they change bioenergetics. This can further impart retrograde signaling, educate cell signaling, epigenetic modifications, chromatin structures, and transcription machinery, and ultimately satisfy cancer cellular and nuclear demands. To maximize the tumor microenvironment (TME), tumor cells remodel nearby stromal cells and extracellular matrix. These changes initiate polyclonality, which is crucial for growth, stress response, and metastasis. Here, we evaluate all the intrinsic and extrinsic pathways drawn by mitochondria in carcinogenesis, emphasizing the perspectives of mitochondrial metabolism in PDAC progression and treatment.

## 1. Introduction

The treatment and detection of several malignancies have made considerable strides over the past ten years, but with significant limitations for pancreatic ductal adenocarcinoma (PDAC), the second-most fatal malignancy by 2030 [1] and today the fourth most common cause of cancer-related mortality [2]. Approximately 20% of patients have localized (possibly curable) illness at the time of diagnosis, 15% have locally progressed (unresectable) tumors, and the remaining 10% have metastatic disease. To make matters worse, even the most potent chemotherapy regimens only significantly increase overall survival by around 11 months and seldom provide long-term progression-free survival (PFS) of >5 years [3]. Therefore, completely new strategies are required to find innovative and more effective PDAC treatments [4].

Metabolic reprogramming increases ATP regeneration by switching to aerobic glycolysis (Warburg effect) for growing energy needs to facilitate tumor growth. Oxidative phosphorylation (OXPHOS) is the primary method by which mitochondria produce ATP. In normoxia, quiescent cells follow this multistep mechanism, which results in the production of 32 ATPs per glucose molecule. However, in a hypoxic tumor microenvironment (TME), anaerobic glycolysis occurs to change glucose into pyruvate and eventually lactate, even in the presence of oxygen (Warburg effect). While OXPHOS produces ATP more quickly, glycolysis produces it at a lower yield: two ATPs per glucose molecule [5]. The Warburg effect with an increased rate of glucose uptake in normally functioning mitochondria in tumor cells contributes to chemoresistance [6,7]. Changing metabolomics in PDAC involving various metabolic pathways has many targetable molecules, and targeting some of them has shown beneficial effects, but still, there is a need for advancements [8,9].

Clinical and basic research aimed at reprogrammed metabolism is now being conducted, reflecting the critical role of reprogrammed metabolism [10]. Tumor cells can maintain redox balance, produce ATP, and engage in biosynthesis due to the comprehensive metabolic ecology of tumors, which encourages carcinogenesis. Cancer cells cause nutritional enrichment, which is a property of complex ecosystems shared by tumors; however, the need for a precise nutrient balance may also be a weakness that may be therapeutically exploited by tumors. Catabolite deprivation may be a selective and successful anticancer treatment method since tumor cells demand greater amounts of catabolite uptake, transport, and use than their normal counterparts. Another approach is to disrupt various metabolic pathways in tumors, and the results show potential by targeting glycolysis and mitochondrial metabolism especially OXPHOS [11]. Compelling findings of metabolic adaptability in PDAC cells suggest that the metabolic characteristics of PDAC may offer promising treatment options [12,13,14,15,16]. The timely screening of PDAC in regular examinations is extremely difficult because of the deeper location of the pancreas. Additionally, the available biomarkers are insufficient to accurately detect PDAC, particularly in its early stages [17].

In this review, we discuss the theoretical aspects of the involvement of mitochondrial metabolism in cancer along with several significant mitochondrial factors that have contributed to PDAC progression, proliferation, stromal interaction, and metastatic dissemination. Next, we focus on the mitochondrial metabolic reprogramming-mediated PDAC progression, epigenetic remodeling, followed by validating their therapeutic significance using the data retrieved from TCGA-cBioportal. Finally, the evidence of mitochondria-based retrograde signaling, extracellular matrix (ECM) dynamics, and immune regulation in PDAC, as well as the therapeutic perspectives of targeting mitochondria in PDAC as an effective therapy alternative are discussed.

## 2. Mitochondrial Metabolism in Neoplastic Transformation

The phrase “neoplastic transformation” typically refers to the alteration of a normal cell into a tumoral precursor that, when immunosurveillance fails, obtains supplemental modifications allowing unrestrained cell proliferation possibilities, propagation, and establishment of remote macrometastases [18]. The three major methods by which mitochondria may be involved in neoplastic transformation include (1) the aberrant intensification of mitochondrial metabolites, such as succinate, 2-hydroxyglutarate (2-HG), and fumarate with prevalent transforming impacts, (2) mitochondrial ROS favoring the accumulation of possibly tumorigenic DNA, and (3) the stimulation of possibly oncogenic signaling pathways. Additionally, mitochondria are crucial for several other processes, including biosynthesis, signaling, apoptosis, cell cycle, cellular differentiation, and cell proliferation, which are all inextricably tied to the development of tumors [19,20].

**Figure 1:**
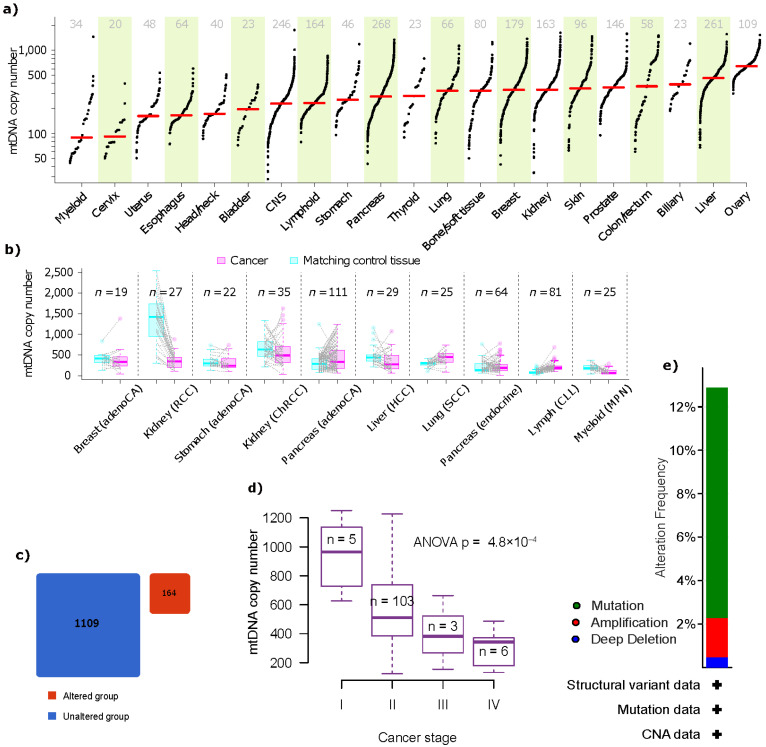
Significance of mtDNA in PDAC progression. (**a**) mtDNA copy number distributions by cancer tissue type. The sample numbers with accessible mtDNA copy number information are indicated on top, with red bars indicating the median mtDNA copy numbers. (**b**) Matched normal tissue samples and tumor samples were compared by copy number. *n* is the number of matched samples of cancer and normal tissue. (**c**) Thirteen mitochondrial genes (*ATP6*, *ATP8*, *COX1*, *COX2*, *COX3*, *CYTB*, *ND1*, *ND2*, *ND3*, *ND4*, *ND4L*, *ND5*, *ND6*) frequency in altered and unaltered pancreatic cancer samples. (**d**) mtDNA copy number and cancer stage in chronic lymphocytic leukemia are correlated. *N* is the number of samples containing information on the stage and mtDNA copy number. (**e**) Frequency of mutation, amplification, and deletion based on 1347 samples gathered from 11 studies. (Data source and picture courtesy: https://www.cbioportal.org/; https://ibl.mdanderson.org/tcma/copy_number.html; https://doi.org/10.1038/s41588-019-0557-x Accessed on 25 January 2023.

A prevalent characteristic of cancer is altered energy metabolism, which has led to a long-standing suspicion that mitochondria may play a role in the development of cancer [21]. The International Cancer Genome Consortium (ICGC) and TCGA projects gathered whole-genome sequencing (WGS) data from 2658 malignancies across 38 tumor types, which were combined by the Pan-Cancer Analysis of Whole Genomes (PCAWG) Consortium, and analyzed mitochondrial somatic mutations and their relationship with nuclear alterations [22]. Increased mtDNA copy numbers for 13 mitochondrial genes were noted in tumor tissues from PDAC patients (*n* = 507), chronic lymphocytic leukemia, and lung squamous cell carcinoma, but decreased copy numbers were seen in patients with kidney clear cell carcinoma, hepatocellular carcinoma, and myeloproliferative neoplasm (Figure 1). The PDAC tissues have shown significant mtDNA copy numbers in a sample size of 111 compared with their matching controls. Moreover, 63% of mtDNA deletion in one PDAC sample (SP76017) was reported. Moreover, mtDNA copy number has shown significant differences in PDAC cancer stages, which may be due to mitochondrial malfunctions upon tumor growth, though further interpretations must be made cautiously. Furthermore, leading mtDNA coverage was shown by PDAC and pancreatic endocrine tumor tissues (12,629.9) after ovary (16,960.4), prostate (14,533.1), and kidney tissues (13,213.0). These results altogether indicate the relevance of mtDNA in PDAC progression relative to other malignancies.

**Figure 2:**
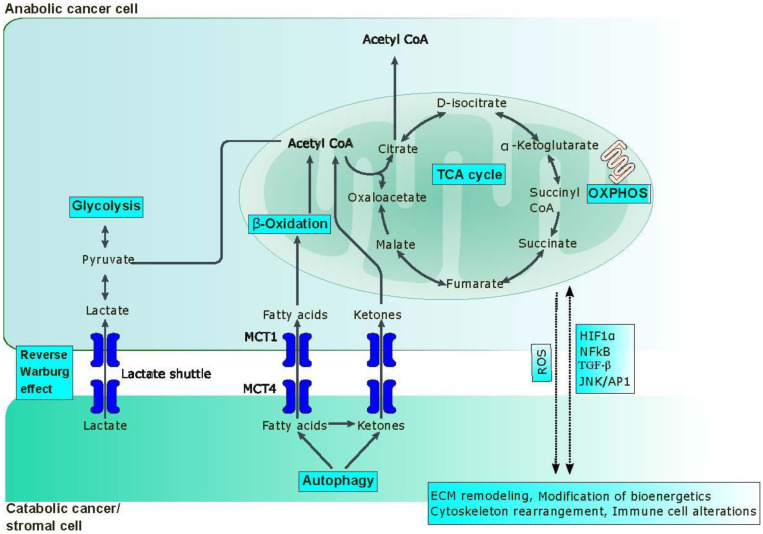
Metabolic adaptations and heterogeneity among cancer cells. Malignant cancer cells possess a high rate of metabolism similar to highly proliferative normal cells. Anabolic cancer cells intake high levels of ketones, fatty acids, and lactate from surrounding catabolic cells in order to replenish the TCA cycle and OXPHOS (reverse Warburg effect). ROS as a metabolic byproduct and HIF1α, NFkB, TGFβ, and JNK pathways, which are activated by the metabolic intermediates, contribute to tumorigenesis through ECM remodeling, altered bioenergetics, cytoskeleton alterations, and immune cell education. TCA, tricarboxylic acid cycle; OXPHOS, oxidative phosphorylation; ROS, reactive oxygen species.

The selective retention of the mitochondrial genome and electron transport chain (ETC) function in malignant tumors in contrast to the benign nature of tumors harboring pathogenic mutations in the mitochondrial DNA highlights the significance of respiration in the development of cancer. Furthermore, oncometabolite produced by mutant TCA cycle enzymes encourages cancer. The strong anabolic metabolism of invasive cancer cells is comparable to that of highly proliferating normal cell types. The TCA cycle, OXPHOS, pentose phosphate pathway (PPP), biosynthesis of hexosamine (responsible for the synthesis of glycosylated compounds), amino acids, and lipids are all supported by the uptake of large quantities of glucose and glutamine by cancer cells [9]. When combined, these mechanisms provide enough cellular building blocks to enable cell growth. Additionally, malignant cells can absorb free fatty acids, lactate, and ketones, which are primarily produced by nearby catabolic cells and may be utilized to restock TCA cycle precursors and fuel OXPHOS (reverse Warburg effect) (Figure 2). The production of adequate amounts of antioxidants, along with the reduced form of glutathione, which is produced by glutathione reductase (with NADPH as the coenzyme) derived from the PPP pathway, is required due to the higher release of ROS in metabolically active cells. Metabolism within tumors can change based on the tumor microenvironment (TME) and the tumor’s proximity to the blood vessels. Nearer to the vascular system, cancer cells benefit from having easier access to oxygen and nutrients. They also create ATP aerobically through OXPHOS and upregulate anabolic pathways, which supports fast multiplication. The stromal cells around the rapidly reproducing cancer cells experience oxidative stress that triggers autophagy and glycolysis, which produces catabolites such as ketones or lactate that are then absorbed by anabolic cancer cells and utilized to generate ATP synthesis and mitochondrial metabolism (reverse Warburg effect). Additionally, inadequate nutrient access forces tumor cells farther from the vasculature and closer to anabolic tumor cell populations to switch to alternate catabolic metabolic pathways, such as autophagy, which allows for more flexibility to satisfy their resource and energy demands [11].

The autophagic clearance of damaged mitochondria, also referred to as mitophagy, is one of the key processes regulating mitochondrial viability and, consequently, restricting ROS generation [23]. It has been shown that, in some situations, the knockdown or deletion of autophagy-related genes (ATG7, ATG5) can enhance tumorigenesis [24,25,26]. Fanconi anemia (FA) genes have also lately been discovered to be involved in mitophagy [27], implying that the tumor inhibitory activity of FA proteins may result from the effective removal of damaged mitochondria which is upregulated with ROS. FA genes are mutated or silenced in a significant portion of human tumors. In addition to enhancing mutagenesis, ROS stimulates other signaling pathways that may lead to cancer, including epidermal growth factor receptor (EGFR) and mitogen-activated protein kinase (MAPK) signaling [18,28]. A number of human cancers have hereditary or spontaneous mutations that impair the enzymes succinate dehydrogenase complex iron sulfur subunit B (SDHB), fumarate hydratase (FH), cytosolic isocitrate dehydrogenase1 (IDH1), and mitochondrial IDH2 [20]. IDH1 and IDH2 usually exhibit gain-of-function mutations resulting in the synthesis of 2-HG, whereas FH and SDHB are typically affected by loss-of-function mutations and are associated with the buildup of succinate and fumarate [29]. Additionally, 2-HG affects the α-ketoglutarate (α-KG)-based prolyl oxidase potential of PHD2 and PHD1, which promotes metamorphosis through a process involving the stability or destabilization of hypoxia-inducible factor 1 alpha subunit (HIF1A) [30,31]. Furthermore, fumarate can cause “succination”, a non-enzymatic post-translational protein alteration that stimulates the neoplastic transcription factor nuclear factor erythroid derived 2 (NFE2), by succinating the kelch-like ECH-associated protein 1 (KEAP1) [32]. It is indeed interesting to note that information sent by neoplastic proteins such as KRAS can also contribute to the buildup of fumarate and succinate rather than just being the product of fundamental mitochondrial abnormalities [33,34]. Similar to this, it appears that the deletion of oncoinhibitory genes such as antigen presenting cells (APC) favors malignant transformation through modifying mitochondrial functions [35]. Most human malignancies cause changes in the mitochondria’s ability to conduct mitochondrial permeability transition (MPT), and these changes are necessary for malignant progenitors to prevent oncogene-based regulated cell death (RCD) [36,37]. The overexpression of BCL2, a cytoprotective factor localized to the mitochondrial outer membrane, may be the source of this change and accompanied manifestation. Many oncogenes besides BCL2 (e.g., MYC, KRAS) promote neoplastic transformation by making the mitochondrial pool more resistant to MPT, often through a process that modifies mitochondrial dynamics. Mitochondria, through producing ROS, contributing to RCD signaling, and assisting anaplerosis, can impart tumorigenesis [38,39,40].

### 2.1. Mitochondria in PDAC Proliferation

Even though tumor cells can obtain enough ATP from glycolysis in vitro under ideal growth settings (which are different from TME of in vivo conditions), mitochondria are necessary for proliferation unless increased levels of pyruvate and uridine are externally provided [41] to make up for aspartate and pyrimidine biosynthesis [42,43]. Indeed, growing tumors exhibit a significant and highly plastic reprogramming of their metabolism. This implicates enhanced glucose absorption, some of which is diverted to the PPP pathway for generating nucleic acids and glutathione reduction, as well as the ability to utilize glutamine either oxidatively for energy synthesis via the Krebs cycle, ETC, or reductively for the synthesis of cholesterol, fatty acids, and the upkeep of oxidative homeostasis through NADPH production. Metabolic adaptability is ensured by the presence of various anaplerotic interconnects focused on mitochondria and the reproducibility of several processes of the TCA cycle [11,44]. Citrate is an important TCA precursor in this regard because of its position as a key juncture between anabolic and catabolic metabolism and thus functions as a significant node of flexibility [45]. Citrate can be used to synthesize cholesterol and fatty acids to endorse the membrane requirement linked with strenuous proliferation, or for acetylation reactions, which control transcription as well as cytoplasmic mechanisms such as autophagy. In addition to bolstering the oxidative mode of the TCA, citrate can also be modified into acetyl-CoA for export to the nucleus and cytoplasm [44,46].

Supporting this, the ATP citrate lyase (ACLY), an enzyme that transforms citrate into acetyl-CoA, is necessary for cancer cells to multiply at optimum rates [47], while normal cells cannot do so because of a metabolic transition from glucose to acetate [48]. In the presence of mitochondrial abnormalities and during hypoxia (based on the α-KG/citrate ratio), reductive glutamine metabolism is the main source of citrate [49,50]. In the latter case, NADPH synthesis (which is essential for lipogenesis and the maintenance of redox equilibrium) is maintained by serine catabolism through serine hydroxy methyltransferase 2 (SHMT2) using reducing equivalents [51,52]. To ensure the production of NADPH from glutamate, intracellular malic enzyme (ME)-1 exerts a similar role in PDACs [13,53]. It is interesting to note that certain human PDACs lack mitochondrial ME2, which makes them reliant on ME3-based NADPH production for growth and survival [54].

### 2.2. Interaction with Stroma

PDAC stroma mainly consists of an extracellular matrix (ECM), cancer-associated fibroblasts (CAF), and vasculature. ECM plays a critical role in tumor progression as well as resistance to therapy by acting as a barrier to drug delivery, vasculature provides nutrients and facilitates progression, and CAFs are involved in active cross-linking with tumor cells to facilitate the growth of the tumor. These three stromal components contribute to low microvascular density, restricted perfusion, and harsh hypoxic environment but aid in tumor survival and growth, and thus are attractive therapeutic targets. Multiple strategies targeting ECM, vasculature, and CAFs have been discussed in the literature, but the results are promising only in pre-clinical trials [8,55]. Due to its collagen I and collagen IV content, ECM is a good source of energy to the growing tumor by providing proline, a digested end product of collagens. Proline taken up by mitochondria is converted to glutamate by proline dehydrogenase 1 (PRODH1) and is a good source of energy entering [56]. CAF activation by paracrine signaling involving the Shh pathway increases mitochondrial activity and proliferation [55].

Malignancies that are progressing exhibit a high level of phenotypic and metabolic flexibility as they develop bidirectional interactions with the TME’s non-transformed elements [57,58,59]. Findings focused on cultured cancer cell lines have generally ignored both of these facets of malignant cells’ biology. Recent in vivo research has shown that the metabolic pattern of malignant cells is influenced by both the TME and the oncogenic driver of the tissue of origin [60,61,62]. A self-renewing subpopulation of cancer cells, called cancer stem cells (CSCs), imparting stemness, is said to exist and be accountable for both localized development and relapse [63]. By enhancing the local accessibility of alanine, which is used by cancerous cells as a source of carbon, PDAC cells induce autophagic responses in tumor-associated fibroblasts (TAFs) that eventually support tumor growth [64]. Upon macropinocytosis, extracellular molecules can also be used by PDAC cells as a carbon source [65], but no mechanisms have yet been identified by which tumor cells can induce non-transformed elements of the TME to secrete proteins for nutrient reasons. Additionally, due to the metabolic commonalities between quickly expanding cells, tumor cells might compete with immune effector cells for nutrients with restricted accessibility, such as tryptophan and glucose [66,67,68]. It is anticipated that such rivalry would affect how likely it is for natural immunosurveillance to slow tumor growth. To avoid competing for glucose, it has been suggested that cancer cells from various tumor locales participate in a metabolic synergy that entails the transfer of lactate produced by glycolysis from hypoxia to the normoxic region. These studies suggest that tumor stroma components may serve as therapeutic targets and altered mitochondrial function should be focused to design novel therapeutic strategies [69,70].

### 2.3. Metastatic Dissemination

The mechanism by which cancer cells can colonize and produce macroscopic lesions at remote places is commonly referred to as metastatic diffusion [71]. Even though macrometastases are frequently thought of as glycolytic elements, this is not necessarily the case [72]. The so-called epithelial-to-mesenchymal transition (EMT), which gives cancer cells more capacity for invasion, is one of the initial changes in the metastatic process. Many mitochondrial metabolites support the EMT [73], particularly fumarate because it can block the production of antimetastatic microRNAs when TET dioxygenases are inhibited [74].

To validate mitochondrial metabolism in PDAC, turning on the Keap1/Nrf2 antioxidant program, the mitochondrial calcium uniporter (MCU) encourages PDAC cell invasion, metastasis, migration, and resistance to metabolic stress. Along the MCU-Nrf2 pathway, the cystine exporter SLC7A11 was discovered to be a therapeutic candidate. SLC7A11 pharmacological inhibitors successfully reduced tumor size and stopped MCU-based metastasis in PDAC. PDAC with high MCU showed greater susceptibility to SLC7A11 suppression in patient-derived xenograft models in vivo and patient-derived organoid models in vitro in comparison to low-MCU tumors. These findings imply that MCU can increase metabolic stress tolerance and stimulate PDAC dissemination in a cystine-associated mechanism [75].

## 3. Metabolic and Molecular Subtypes of PDAC

The metabolic profiles of PDAC can be correlated with its metabolic subtypes, for instance, glycolytic tumors are associated with aggressive squamous or basal profiles, and the lipogenic subtypes shows similarity with classical or progenitor subtypes. The molecular profiles of PDAC are significantly marked by the mutated *KRAS*, *TP53*, *CDKN2A*, *CTLA4*, *PD1*, *KDM6A*, and *SMAD4* [76]. Based on the molecular profiling and gene expressions, the PDAC tissues are categorized into classical (better prognosis after resection), exocrine-like (specificity not determined), quasi-mesenchymal (poor prognosis) [77,78], and basal (similarity with basal tumors) [79]. Moreover, the transcriptome profiling has classified PDAC tissues into squamous, pancreatic progenitor, immunogenic, and aberrantly differentiated endocrine exocrine (ADEX) (well-reviewed here [80]). Retrospective meta-analysis using whole transcriptome data of PDAC patients indicated six different molecular subtypes, where L1, L2, and L6 corresponded to tumor-specific subtypes and L3, L4, and L5 correlated with stromal-specific subtypes. Interestingly, L1, L2, and L6 (tumor-specific) have shown to be enriched with metabolic gene expression. While the L2 subtype maximized glycolysis gene sets and downregulated lipid metabolism, L1 tumors upregulated glycolytic and lipogenic genes. L6 enhanced the activation of genes related to digestive enzymes and protein metabolism [81].

The glycolytic and lipogenic attributes of PDAC corresponding to their molecular profiles were studied by Bryant et al. [82] and Cornell et al. [83]. Upregulation of glucose and lactate transporters such as GLUT1, MCT1, and MCT4 was mediated by PDAC driver mutations, such as *TP53* and *KRAS*. This enhanced lactate dehydrogenase (LDH)-A, hexokinase (HK)-1 and HK2 expression in PDAC cells for the glycolytic flux even when glucose is scarce [82]. Cancer cells can enhance cholesterol for the recruitment of key oncogene receptors and ligands through the mevalonate pathway or by the intracellular bidirectional trafficking of low-density lipoproteins (LDL) and LDLR. High LDLR expression is correlated with a greater likelihood of tumor recurrence in PDAC patients, whereas cholesterol biosynthesis is linked with a more differentiated phenotype (classical subtype) [83].

Furthermore, in order to identify four metabolic subgroups of PDAC, namely glycolytic, quiescent, cholesterogenic, and mixed, Karasinska et al. examined the gene expression involved in glycolysis and cholesterol biosynthesis in clinical samples. While the quiescent group had a low metabolism rate, one of these pathways was made more active by cholesterogenic and glycolytic subtypes. The *KRAS* and *MYC* oncogenes were amplified in the glycolytic subtype, which also had the lowest activity of mitochondrial pyruvate transporters MPC1 and MPC2. Additionally, the cholesterogenic group exhibited the longest median survival, while the glycolytic group had a dismal prognosis. Furthermore, both the glycolytic and cholesterol production pathways were highly active and enriched in the mixed subtype. The categorization of metabolism by Karasinska et al. was juxtaposed with earlier molecular markers. The majority of the quiescent group belonged to the classical subgroup, and it had the highest frequency of ADEX and instances that resembled exocrine function. The basal, quasi-mesenchymal subtypes and squamous were connected with the glycolytic subtype. Moreover, the cholesterogenic group exhibited the largest number of pancreatic progenitor subtypes but the lowest amount of poor prognosis markers [84].

## 4. Metabolic Alterations and Epigenetic Reprograming

The epigenome and cellular metabolism interact with molecular and genetic factors that control cancer in a bidirectional manner. Metabolites can regulate chromatin or protein acetylation (e.g., c-MYC, HIF-1α, GAPDH, PKM2, PEPCK, 6PGD, ACLY, LCAD, and BCAT2), histone succinylation, butyrylation, lactylation, phosphorylation, citrullination, and itaconation, and further contribute to cancer progression [85]. Epigenetic aberrations play crucial roles regulating the redox balance of cancer cells by modulating the expression of metabolic genes [86]. Contrarily, metabolic flow influences energy generation and macromolecule biosynthesis, which influences how epigenetic regulation is carried out [87]. For instance, c-MYC regulates glucose, glutamine, and serine homeostasis at the transcriptional level and promotes succinate dehydrogenase complex subunit A (SDHA) acetylation by encouraging sirtuin3 degradation, which results in SDHA deactivation and succinate accretion. Succinate levels rise, histone demethylases are inhibited, H3K4 trimethylation is triggered, tumor-specific genes are expressed, and followed by tumor progression [88]. The regulation of DNA methylation and histone H3K9 that is supported by the 6-phosphogluconate dehydrogenase (6PGD)-mediated oxidative PPP pathway during the evolution of PDAC encourages the transcription of N-cadherin and N-cadherin-mediated metastasis [89]. By connecting metabolic and epigenetic changes, SET domain-containing 2 (SETD2), a histone lysine methyltransferase, integrates enhancer of zeste homolog 2 (EZH2) and the AMPK signaling pathway to limit prostate cancer spread [90]. In diffuse intrinsic pontine gliomas, the H3.3K27M mutation causes a decrease in global H3K27me3 through a variety of mechanisms, including abnormal PRC2 interactions or constrained H3K27me3 spreading [91]. Moreover, the *KRAS*-mutant pancreatic acinar cells are supported in their proliferation by the AKT–ACLY axis, and inhibition of AKT lowers histone acetylation and inhibits acinar-to-ductal metaplasia (ADM). Without obvious metabolic problems, pancreas-specific ablation of ACLY suppresses ADM and pancreatic carcinogenesis. It was discovered that ACLY is a potential substrate for caspase-10, which cleaves at the conserved Asp1026 location. Increased caspase-10 reduces intracellular lipid levels and suppresses GCN5-based histone H3 and H4 acetylation by ACLY cleavage under metabolic stress conditions, such as glucose deprivation. This inhibits the expression of tumor-related proliferative and metastatic genes as well as tumor growth [92]. CREB-binding protein (CBP) acetylates the rate-limiting enzyme of BCAA metabolism, branched-chain amino acid transaminase 2 (BCAT2), at position K44. This post-transcriptional modification of BCAT2 encourages its destruction and inhibits BCAA catabolism and the spread of pancreatic cancer [93]. Loss of LKB1 or STK11 and stimulation of KRAS work in concert to enhance serine metabolism and further cancer progression. The increased de novo serine biosynthesis pathway encourages DNA methylation in LKB1-deficient cells. Moreover, loss of LKB1 reduces retrotransposon expression and DNA methylation [94].

## 5. Mechanism Underlying PDAC Progression via Metabolic Reprogramming

Mitochondria modulate the growth of the tumor, its capacity to metastasize, and finally, the activation of chemoresistance pathways. The majority of cancer cells possess mutated mtDNA, and the signal crosstalk between mitochondrial and nucleus signaling enhances cancer progression through rewiring the common metabolic pathways [95]. Furthermore, mitochondrial DNA (mtDNA) mutations, mitochondrial enzyme defects, alterations in bioenergetics, ROS production, and mitochondrial redox biology contribute to metabolic reprograming in cancer [96]. A broad range of malignancies have been documented to have germline and somatic mtDNA alterations. These malignancies include colon cancer, renal adenocarcinoma, head and neck tumors, ovarian tumors, thyroid tumors, breast tumors, prostate and bladder cancer, and neuroblastomas [21,97,98]. Although technological and explanatory errors were frequent in mtDNA-based mutational studies [99,100,101], the discovery of mtDNA mutations that are harmful in tumor tissue, such as an intronic deletion or the frequent tRNA-Leu (UUR) A3243G MELAS mutation [100], validates the importance of pathogenic variants in malignant transformation.

Based on the meta-analysis of many mtDNA alterations linked to cancer, several of these alterations blatantly impede OXPHOS in cancer cells. Nevertheless, a sizeable part of the observed variants is identical nucleotide alterations which have already been identified in human groups as early adaptive mtDNA variants [102]. These so-called “connections” with cancer can range from true cancer cell mutations to glaring misconceptions of ancient polymorphisms [103]. As a result, there could be two types of alterations in the mtDNA of cancer cells: those that affect OXPHOS and promote tumorigenesis and those that help cancer cells adapt to shifting bioenergetic environments [21].

**Figure 3:**
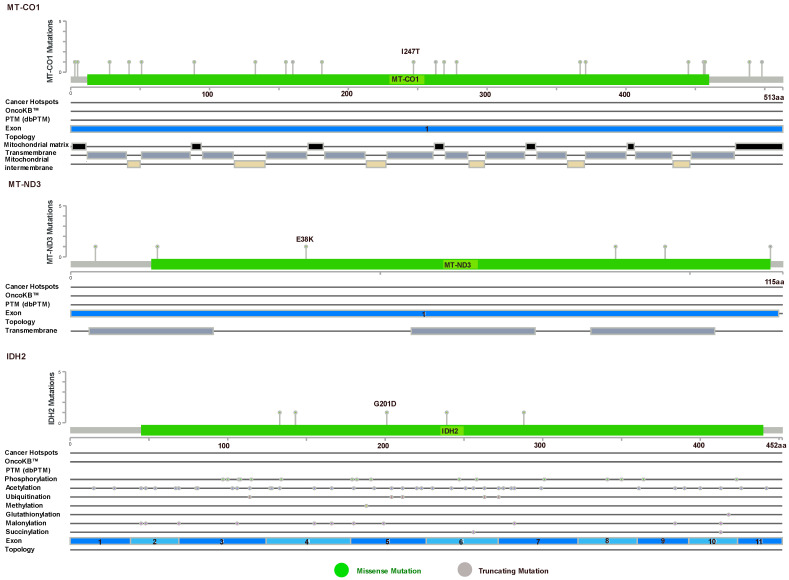
Lollipop diagram of mtDNA MT-CO1, MT-ND3, and IDH2 in PDAC. (Data source: https://www.cbioportal.org/ accessed on 10 January 2023 [105].)

In a recent investigation, the mtDNA genotype and pancreatic cancer phenotype were linked using patient-derived cell lines. In 12 patient-derived pancreatic cancer cell lines, the scientists examined the mtDNA and over 1000 nDNA encoding metabolic enzymes and mitochondrial proteins. They found 24 somatic variations in mtDNA, and 18 mutations in the nDNA. In light of these somatic alterations, evaluation of metabolic function revealed alterations in metabolism that were congruent with mitochondrial impairment. Notably, the bulk of somatic mtDNA alterations was discovered in complex I subunits and non-coding regulatory areas. A few mutations were also reported in CYTB and COX1 [104]. To examine the clinical significance of majorly studied mitochondrial genes such as MT-ND3, MT-CO1, TFAM, and PDAC progression, we extracted and validated the TCGA-cBioportal data considering 1174 samples [105]. The result indicated five missense and one truncating mutation in MT-ND3, eighteen missense and three truncating mutations in MT-CO1, and eight missense mutations in IDH2, but no specific mutations were seen in TFAM (Figure 3) (Table 1).

Glucose, amino acids, and lipid metabolisms are altered in cancer cells including PDAC as an adaptation for meeting bioenergetics [9]. In mitochondria, ETC converts glucose into CO_2_ and ATP. For anabolic processes such as the production of amino acids, ribose, lipids, and intermediates to glycosylation, cancer cells require more glucose [106]. Mutant KRAS controls glucose transporter 1 (GLUT1) in PDAC cells, which enhances glucose absorption [12,107] and provides intermediates for biosynthetic pathways. To encourage glycolysis, mutant KRAS also increases the activity of the enzymes hexokinase 1/2 (HK1/2), lactate dehydrogenase A (LDHA), and phosphofructokinase 1 [12,108]. Additionally, to boost the activity of glycolytic enzymes and sustain cytosolic ATP, mutant KRAS works in conjunction with other pathways and the hypoxic TME [109,110,111,112]. KRAS4A engages with HK1 in mitochondria and actively controls HK1 in conjunction with the transcriptional activation of glycolytic enzymes and glucose transporters [113]. Uridine diphosphate-N-acetylglucosamine is produced when mutant KRAS stimulates the hexosamine biosynthetic pathway (HBP) for glycosylation [12,114,115]. A further anabolic mechanism by which mutant KRAS enhances the glucose flow in the mitochondria is the PPP pathway. The oxidative and nonoxidative stages of this route, which is significant for creating nucleotide synthesis precursors, are separate from one another. In the oxidative phase, two NADPH molecules are concurrently synthesized when ribulose 5-phosphate is transformed from glucose 6-phosphate. NADPH is then employed for redox regulation and fatty acid production. The processes that generate ribose 5-phosphate (R5P) for nucleic acid biogenesis make up the nonoxidative PPP phase. Additionally, MYC overexpression causes mutant KRAS in PDAC cells to become reliant on the nonoxidative PPP phase [116]. By maintaining HIF1, MUC1 further promotes anabolic glucose metabolism [117,118,119,120]. This unequal dependence on the nonoxidative phase may be a metabolic susceptibility of pancreatic cancer since normal cells create R5P in the oxidative phase. These glycolytic alterations begin to develop at the onset of precancerous lesions and continue as the tumor grows [8]. Increased lactate dehydrogenase synthesis produces lactate, which is then transferred outside the cell by monocarboxylate transporters (MCTs) to maintain glycolysis, and is ultimately modulated by KRAS. Additionally, pyruvate provides carbon to the TCA cycle. Moreover, the TCA cycle is powered by glutamine, which is imported by SLC1A5 and processed to glutamate by glutaminase 1 (GLS1), which is indirectly assisted by KRAS. Interestingly, mutant KRAS enables ME1 to redirect TCA cycle intermediates toward the synthesis of asparagine, NADPH, and aspartate, and further the bioenergetic pathways [121].

Furthermore, in PDAC cells, the number of amino acids exchangers are dramatically increased to meet the metabolic requirements of growing tumors [122,123] by facilitating carbon and nitrogen needed for the formation of macromolecules. Amino acid glutamine is crucial for tumor cells [124]. The TCA cycle is continually replenished by glutamine to yield intermediate reactions and substrates for macromolecular production. The TCA cycle is refilled by the conventional glutamine route, which transforms glutamate produced from glutamine into α-KG. This process results in the production of NADH as well as the precursors to lipids and biomolecules. Purines and pyrimidines can both be found in significant amounts in glutamine [125,126]. This suggests that reprogramming of amino acids not only facilitates the production of ATP but also nucleotide metabolomics. It is possible to employ glutamine as a substrate for the production of nucleotides as well as glutamine-derived aspartate [127]. Glutamine is also used by PDAC cells to establish redox equilibrium [13]. In this mechanism, glutamine has two functions. First, glutamine is a source for the generation of glutathione, which functions as an antioxidant to shield cells against free radical damage. Second, through a non-canonical glutamine metabolism route, mutant KRAS encourages the creation of electron carriers in the form of NADPH. By the enzyme aspartate aminotransferase (GOT2) in the mitochondria, glutamate generated from glutamine is changed into aspartate. When this aspartate enters the cytoplasm, it is processed by the intracellular aspartate aminotransferase, malic enzyme (ME1), and malate dehydrogenase, which leads to the synthesis of NADPH [128]. PDAC cells can retain the redox balance by accelerating anaplerotic glutamine utilization during stress. Along with the contribution of KRAS to metabolic alterations, p53 is critical in remodeling glutamine and glucose metabolism to generate α-KG [129]. Accumulated α-KG proceeds via chromatin remodeling and has a tumor-suppressing impact through p53. In PDAC cells, the metabolism of additional amino acids is altered. Under nutrient-restricted circumstances, proline produced from the ECM enhances PDAC cell survival [56]. When there is a shortage of biofuels, PDAC cells consume ECM collagens through processes that are both reliant on and independent of macropinocytosis. Through the preservation of nutrient and oxidative equilibrium, cysteine is also crucial for promoting PDAC cell survival [130]. The cystine/glutamate transporter (xCT), which may be a viable therapeutic target, is vital for maintaining cysteine equilibrium. Additionally, the diagnostic potential of PDAC is linked to a rise in plasma branched-chain amino acids, which may be caused by an accelerated degradation of tissue protein [131,132].

Lipid metabolism is crucial for the development of PDAC [133]. Many fatty acid and cholesterol synthesis-related activities, as well as cholesterol absorption, are markedly increased in PDAC cells [134,135]. In PDAC cells with a p53 alteration and loss of heterozygosity, sterol O-acyltransferase 1 (SOAT1) prevents cholesterol feedback prohibition by unesterified cholesterol via promoting the mevalonate pathway. The expansion of healthy cells with wild-type p53, in contrast, is unaffected by SOAT1 suppression, indicating possible therapeutic potential [136].

## 6. Mitochondrial Retrograde Signaling in PDAC

Recent progress in extracellular vesicle (EV) research has shown their relevance in cancer progression through bidirectional trafficking, including antero and retrograde signaling. Mitochondria-derived circulating EVs from PDAC have shown to possess mtDNA mutations in respiratory complex I (*ND1-6*) and III (*CYTB*) genes. Moreover, ncDNA such as D loop and *RNR2* exhibited 15.2% mutation among other EV-based mtDNAs in PDAC [137]. Essentially, the EVs are essential mediators for establishing biological crosstalk amongst cancer cells and numerous cellular constituents within the TME for fostering carcinogenesis by dispersing their cargos [138]. It has been demonstrated that EVs encourage PDACs’ proliferation, angiogenesis, chemoresistance, and migration [139,140]. Exosomes obtained from PDAC patients have been shown by Tang et al. and demonstrated their role in cell proliferation and migration using MiaPaCa-2 and AsPC-1 cells [141].

Moreover, alterations in mitochondrial genes can affect cellular bioenergetics in ways that are crucial for tumorigenesis because mitochondrial gene variations are frequent in cancer and mtDNA-encoded OXPHOS genes are necessary for cancer cell survival and expansion. Nonetheless, for *SDH* flaws, HIF1 signaling is activated; for *FH* faults, NRF2 signaling is changed; for *IDH1* and *IDH2* alterations, redox signaling is changed; and for all four genes, chromatin methylation and the epigenome are changed. Therefore, retrograde signaling, a method of reprogramming the nucleus by cytoplasmic constituents, must be employed by mitochondria [96].

Alterations in mitochondrial ROS generation, as well as Ca^2+^ signaling and redox regulation, have been linked to shifts in retrograde signaling. Reversibly treating cells, for instance, mouse myoblast C2C12 cells, with either EtBr (to lower the mtDNA concentration 50–80%) or the mitochondrial uncoupler carbonyl cyanide m-chlorophenyl hydrazone (CCCP), has demonstrated the significance of mitochondrial Ca^2+^ modulation in retrograde signaling. The electrical potential of the mitochondrial inner membrane is reduced by both procedures. In transplantation studies, these procedures change the C2C12 cells from becoming nontumorigenic to having malignant growth patterns [142]. The energy demand for mitochondria to receive Ca^2+^ is decreased when the mitochondrial membrane tension decreases, increasing the intracellular Ca^2+^ level. The nuclear relocation of the REL-p50 heterodimer is brought about by the activation of calcineurin by cytosolic Ca^2+,^ which then triggers the activation of IkB-dependent NFkB. REL-p50 then forms a nuclear enhanceosome with CREB, NFAT, and C/EBP [143]. Furthermore, calcineurin stimulates the insulin-like growth factor 1 receptor (IGF1R), which stimulates AKT by way of PI3K. When phosphorylated and activated, heterogeneous nuclear ribonucleoprotein A2 (HNRNPA2) functions as the transcriptional co-activator of enhanceosome, which is supported by the accession of phospho-AKT into the nucleus. Almost 120 nDNA genes can be transcriptionally upregulated as a result of these alterations, including genes responsible for apoptosis (*BAX*, *BID*, *BCL-X*, *BAD*), Ca^2+^ regulation (calreticulin, *RYR1*, calsequestrin), tissue invasiveness and tumorigenesis (*TGFΒ*, *P53*, *AKT1*), and glucose metabolism (*IRS1*) [144,145,146,147].

## 7. Mitochondria-Assisted ECM Dynamics in PDAC

The TME is composed of several elements that surround tumors. In addition to ECM, which offers the resident cells biochemical and mechanical support, TME is made up of many cell types, including endothelial, immunological, and fibroblast cells [148]. TME has been discovered to be hypoxic and devoid of nutrients because of the rapid tumor development rate and the constrained blood supply [149]. Consequently, to survive, tumor cells might change their metabolism and develop nutrition-scavenging techniques. Moreover, nutritional signaling and lipid and glucose metabolism can be controlled by the pressure produced by cell–ECM contact [56,150,151]. The supply of nutrients from external sources can potentially have an impact on mitochondrial dynamics. Previous studies have demonstrated that tumor cells maintained in Hank’s Balanced Salt Solution (HBSS, a low-glucose medium) had the propensity to maintain their mitochondria in a linked, protracted configuration. The silencing of DRP1 (mitochondrial fission protein) was caused by the protein kinase A (PKA)-based phosphorylation of *DRP1* at Ser637. In turn, this led to a transition in the metabolism of cancer cells from glycolysis to mitochondrial OXPHOS, which aided in cell survival [152]. Increasing data point to a connection between mitochondria and ECM and suggest that mitochondria are capable of detecting changes in the TME, including alterations in the ECM’s structure and rigidity.

ECM formation is significantly enhanced throughout pancreatic cancer growth, and it has been demonstrated that ECM firmness in this setting affects mitochondrial dynamics. To meet the ATP requirement brought on by cytoskeletal remodeling and cell migration, PDAC cell lines’ mitochondria lengthen in the stiff ECM and congregate in invasive protuberances [153]. The phosphocreatine (pCr)–creatine kinase (CK) system, which allows creatine to be phosphorylated into phosphocreatine, may recycle ATP and maintain local ATP gradients. The cytoplasmic creatine kinase B-type (CKB) is responsible for catalyzing this process, and it has been discovered that the biomechanical cues produced by rigid ECM stimulate its production in a way that is reliant on integrin and YAP signaling, which further enhance OXPHOS activity and mitochondria fusion.

The equilibrium between apoptosis and survival is one of the key factors that cells must regulate. ECM dissociation leads to cell death, also called anoikisis [154]. The outer mitochondrial membrane’s permeabilization can secrete a variety of proteins, such as cytochrome c, which activate apoptosis via caspases [155]. After ECM separation, pancreatic cancer cells’ mitochondrial activity may change as a result of mitochondrial depolarization as well as the secretion of proapoptotic agents, which eventually causes necrosis.

Pancreatic cancer cells possess enhanced survival upon binding with ECM proteins. After mounting pancreatic cancer cells on fibronectin or laminin, the mitochondrial deregulation was suppressed because these ECM proteins raised the mitochondrial membrane potential and prevented the release of the proapoptotic molecules including cytochrome c and Smac/DIABLO [156]. Further, ECM-detached cells can trigger the receptor-interacting protein kinase 1 (RIPK1), which then activates mitophagy through mitochondrial phosphatase phosphoglycerate mutase 5 (PGAM5), causing the production of nonapoptotic cell death and ROS release [157]. However, the human protein atlas indicates that RIPK1 production is elevated in the vast majority of cancer types. As a result, perhaps tumor cells can avoid the non-apoptotic cell death caused by RIPK1 or mitophagy and can aid in cancer cells’ survival by removing damaged mitochondria [157]. Moreover, OXPHOS can control ECM remodeling factors, including matrix metalloproteinase (MMPs) and their inhibitors such as tissue inhibitors of proteases (TIMPs), as shown in osteosarcoma [158].

## 8. Mitochondria in Immune Regulation

Mitochondrial metabolic processes have significant impacts on immunity [159]. Valle et al. showed by switching the in vitro carbon source from glucose to galactose that the PDAC cells were able to use OXPHOS, which led to upregulated immune evasion properties, enrichment of CSCs indicated by higher expression of pluripotency and CSC biomarkers, increased transformation potential, induced but reversible quiescence, enhanced OXPHOS function, and increased invasiveness [4]. Further, the interpretation of ligands and receptors involved in the immune silencing of T cells (PD-L1) revealed highly elevated invasion, migration, metastasis (CD155 and CD206), anti-phagocytic function (CD47), PaCSC immune evasion, and autophagic vesicles.

**Figure 4:**
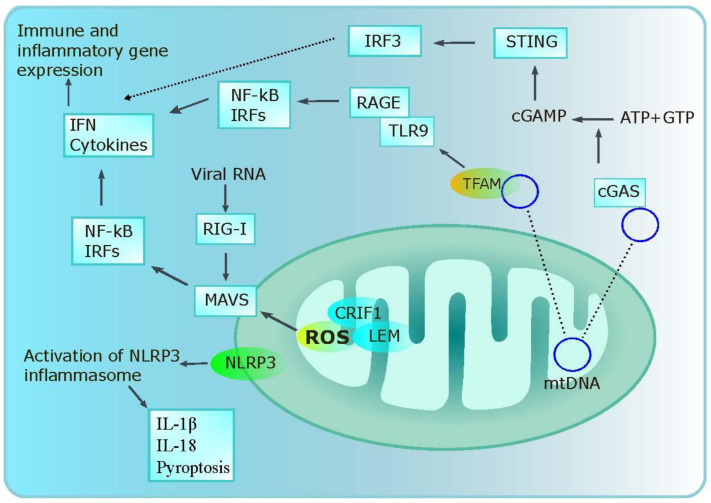
Mitochondria in immune regulation. Mitochondrial ROS through MAVS and NLRP3 enhance cytokine secretion. NLRP3 assists pyroptosis while the NFkB-based MAVS–cytokine axis enhances inflammatory gene expression. mtDNA and cGAS activate STING-based IRF3 and contribute to immune gene expressions. TFAM and mtDNA activate NFkB and IRFs to further immune responses. MAVS, mitochondrial antiviral-signaling protein; IRF, interferon regulatory factor; NLRP, NLRP domains containing protein; TLR, toll-like receptor; cGAS, cGMP-AMP synthase; RAGE, the receptor for advanced glycation end products.

Immune and inflammatory gene expression is promoted by mitochondrial antiviral-signaling protein (MAVS) via interferon regulatory factor (IRFs) and NF-kB once it is triggered by the viral RNA sensor retinoic acid-inducible gene (RIG)-1. Additionally, mitochondrial ROS may trigger MAVS independent of RNA. Cardiolipin, which is projected to the outside membrane when mitochondria are depolarized, is required by the NLRP domains containing protein (NLRP)-3 inflammasome and also transmits information from the outer mitochondrial membrane. Comparable to MAVS, NLRP3 also reacts to mitochondrial ROS and can affect mitochondria, which encourages the production of more ROS. IL-18, IL-1β, and pyroptosis are all induced by NLRP3. Additionally, mtDNA can stimulate NLRP3 and is detected by Toll-like receptor (TLR)-9, which causes the production of inflammatory and immunological genes. Last but not least, MAVS encourages NLRP3 oligomerization at the mitochondria, ensuring innate immunity modulation [159]. Moreover, a stress signal that is generated from the mitochondria can activate the IFN gene stimulator (STING pathway). STING is activated by cGAMP, which is created when mtDNA interacts with cGMP-AMP synthase (cGAS). IFN and additional IFN-stimulated genes can then be expressed as a result of IRF3. A protein known as mitochondrial transcription factor A (TFAM) binds to mtDNA. By attaching to the receptor for advanced glycation end products (RAGE) and TLR9, TFAM functions as a threat signal and improves the plasmacytoid dendritic cell (pDC) response after cell injury or necrosis [160] (Figure 4).

OXPHOS is significantly modulated in M1 (LPS or LPS plus IFN-γ activated) and M2 (IL-4 activated) macrophages, which is related to their varied functions in the immune reaction. M1 macrophages are more inflammatory and play a key role in the removal of microbial infections. Inflammation and tissue healing are regulated by M2 macrophages [161]. It is well known that activation of M1 macrophages and DCs causes an increase in glycolysis and PPP pathway while decreasing OXPHOS [162,163]. NO synthesis from arginine leads to mitochondrial collapse or ATP reduction in DCs and macrophages following activation with LPS and other pathogen-associated molecular patterns (PAMPS) [164]. By nitrosylating proteins containing iron and sulfur, such as complex I, II, and IV of ETC, nitric oxide (NO) prevents electron transport and consequent ATP synthesis [165]. The reprogramming of mitochondria to produce mtROS, signaling molecules necessary for the formation of an adequate immune response, from complex I is one effect of the reduction in ATP synthesis via OXPHOS in LPS-based macrophages [166]. It is crucial to remember that LPS-treated macrophages need complex I activity and glycolysis to provide the proper immunological response. Thus, the activation of IL-1 in response to LPS is compromised by both the reduction in glycolysis with 2-deoxyglucose [163] and the restriction of complex I activity with metformin [167]. Metabolites produced by non-immune cells that are present in the TME can also impact immune cells’ capacity to perform OXPHOS and glycolysis and change their morphology. By polarizing TAM to M2-like phenotype in the TME through a process that depends on HIF-1α, lactate generated by tumor cells may accelerate the development of cancer [168]. Lower levels of lactate are found in vivo in tumors that lack the gene-encoding pyruvate kinase M2. These tumors also exhibit reduced levels of the M2 marker arginase and are considerably smaller than tumors with normal gene expression. By altering the biochemistry of DCs in a lactate-dependent way, mesenchymal stromal cells (MSCs) can change the phenotypic and functioning of these cells. This impact is reversed with the introduction of a lactate dehydrogenase inhibitor. By promoting M2-like features in monocytes and enhancing their antigen-presentation potential, lactate generation by MSCs inhibits the development of monocytes into DCs and favorably polarizes CD4+ T cells to TH2 cell phenotype. Additionally, lactate released from MSCs reduces OXPHOS and mitochondrial activity in developing DCs. Given that M2 macrophages prefer OXPHOS, this discovery is rather perplexing; nonetheless, lactate generation may potentially produce a HIF-1α-based increase in glycolysis, although further confirmatory studies are needed [169].

## 9. Targeting Mitochondrial Metabolism in PDAC

Treating tumors based on their distinct genetic profiles has proven to be difficult due to molecular alterations and tumor heterogeneity. Therapy based on this shared characteristic may prove to be a more effective anticancer approach than treatment based on the comprehensive and highly variable genetic makeup of cancers since targeting the genomic sequence has proven challenging and subjected the patients to significant toxicity when taken in conjunction with the vast heterogeneity that makes most tumors biologically distinctive [11]. Highly malignant tumor cells exhibit tolerance to cell death, which is linked to aberrant metabolism [170]. In fact, diverse cell populations with varied metabolic profiles have been found in a mouse model of pancreatic cancer; the tumor cells which are most resilient to apoptosis depend on OXPHOS [171]. Additionally, there is metabolic diversity between the tissues of the host and the tumor. Accordingly, therapies that target tumor metabolism possess the potential to enhance patient outcomes; however, due to dose-limiting toxicity, the production of medications targeting metabolic activities is complicated by the fact that normal tissues commonly activate pathways that are elevated in cancer. Defining the metabolic variations among cancer cells and healthy cells better and using medicines that take advantage of these differences may enhance the effectiveness of cancer treatment.

It is well known that the mitochondrial activity in stem cells differs from that in their mature counterparts. When stem cells divide asymmetrically, it happens frequently that one new cell will maintain stem cell characteristics while the other differentiates, creating a hierarchy of stem cells. Asymmetric mitochondrial allocation results upon stem cell replication, with older mitochondria with reduced membrane potential being allocated to the daughter cell that would differentiate [172]. It has been demonstrated that pancreatic CSCs depend on OXPHOS for survival [171]. Additionally, CSC characteristics must be maintained, which requires mitochondrial metabolism [173]. The metabolic profile of CSCs and the impact of mitochondrial effectors on CSC populations should be investigated as they may serve as a therapeutic target in PDAC.

Potential anti-cancer medications have been investigated to block OXPHOS (Table 2). Inhibiting NADH-coenzyme Q oxidoreductase (complex I) and Q-cytochrome c oxidoreductase (complex III) interfere with OXPHOS and are arsenic trioxide and metformin, respectively [174,175]. Arsenic trioxide selectively causes mitochondrial ETC leakage to accelerate, which disturbs mitochondrial respiration [176]. Arsenic trioxide is being researched for use in different cancer types, including PDAC [174]. Metformin, a drug licensed to treat type 2 diabetes mellitus, decreases hepatic gluconeogenesis. Nonetheless, concern about the utilization of metformin as an anticancer drug was ignited by these epidemiological results. In fact, metformin has shown preclinical anticancer efficacy in vitro and in animals, and clinical trials have produced biomarker evidence of its antiproliferative effects [177,178]. Since certain cancers are exposed to 10–40 times less glucose than normal tissues, this observation may have physiological significance [179,180]. It was demonstrated that CSCs, cells with low glucose uptake, and cells having mutations in the OXPHOS complex I [181,182,183,184] are highly cytotoxic to metformin. Metformin’s anticancer action has so far been shown in clinical studies to be effective in treating individuals with breast, prostate cancer, and endometrial cancers, but not in pancreatic cancer [185,186].

Complex I is the most commonly mentioned mitochondrial respiratory complex in pancreatic cancer and other malignancies. This revelation that biguanide metformin, a complex I inhibitor, improved outcomes in diabetic individuals suffering from PDAC sparked attention to the topic [188,189,190]. Interestingly, this positive influence was statistically significant in the retrospective analysis by Sadeghi et al. [189] only in patients who had the non-metastatic disease. Metformin with chemotherapy did not, however, increase patient survival in clinical studies for PDAC patients [186,191]. This occurs in pancreatic cancer as well as other malignancies [192,193]. Mitochondrial Complex I suppression is the main mode of action of the pharmacological agents phenformin and metformin to prevent tumor development. Additionally, many studies revealed that synthetic drugs cause mitochondria to produce less ATP and use less oxygen, which promotes the breakdown of carbohydrates to make up for inefficient mitochondrial metabolism [194,195,196]. Accordingly, Andrzejewski et al. [194] revealed that metformin produces lower glucose utilization through the TCA cycle in isolated mitochondria or whole cells and that culture in reduced glucose settings resulted in increased sensitivity to metformin. The cumulative results of this research show that metformin directly affects mitochondrial metabolism and that tumor cells fight this effect by increasing glycolysis, which ultimately results in increased lactate generation. More significantly, in vivo testing revealed that metformin has a direct effect on mitochondrial complex I [192]. Candido et al. [197] mixed a suboptimal dosage of metformin with the rapamycin (mTOR inhibitor) considering the hypothesis that metformin has an effect through AMP-activated protein kinase (AMPK) activation, which further inhibits mTORC1 (leading to autophagy). In two PDAC cell lines (BxPC-3 and MIA PaCa-2), but not in the ASPC-1 cell line, this study found that metformin decreased the IC50 of rapamycin. Astonishingly, administration with metformin alone had no impact on growth in BxPC-3 or MIA PaCa-2 cells, despite the medication’s relatively low (5M) concentration. In addition to mTORC1, AMPK activation inhibits the Raf/MEK/ERK and PI3K/PTEN/Akt/mTORC1 pathways. In MIA PaCa-2 and BxPC-3, metformin also enhances the effects of a PI3K/mTOR inhibitor. Considering the actions of PDAC stroma, another novel method to prevent PDAC carcinogenesis has been developed. Metformin was shown by Duan et al. [198] and Qian et al. [199] to diminish the desmoplastic stroma, which increased chemotherapy’s anticancer effects. Whatever their mode of action, metformin and phenformin have demonstrated potent potential anticancer activity in vitro and in vivo in many malignancies, including PDAC [200,201]. Because phenformin has a greater permeability and hydrophobicity, its anticancer effect is more potent. Synthetic drugs are a great choice to manage pancreatic cancer, according to nearly all contemporary and older evidence. Additionally, the current strategy involves using combination treatment, typically with chemotherapy, glycolytic inhibitors, or other substances [197,198,199,202,203]. Intriguingly, Gravel et al. [204] showed that dietary restriction of glycine and serine potentiates the anti-cancer effects of phenformin in colon adenocarcinoma allograft models. To impose the metformin-antitumoral effect at pharmaceutical dosages, several studies propose the usage of metformin analogs that have a more effective anticancer effect [205]. Last but not least, various clinical studies have been conducted to examine the effects of metformin on PDAC.

Clinical studies utilizing an antagonist of pyruvate dehydrogenase and α-ketoglutarate dehydrogenase enzymes are presently being conducted on a more pertinent TCA cycle target in PDAC [206]. Devimistat, also referred to as CPI-613, is a specific cancer drug that is now being tested in phase II clinical trials for people with pancreatic cancer that cannot be surgically removed (NCT03699319). The goal is to provide Devimistat together with a modified FOLFIRINOX (mFOLFIRINOX) to participants. Phase III research of mFOLFIRINOX with Devimistat in patients with metastatic PDAC is also ongoing (NCT03504423). Patients with metastatic PDAC had a 61% response rate to Devimistat coupled with mFOLFIRINOX in a modest cohort phase I study [207]. The combined regimen was also well tolerated and secure, prompting additional research into this treatment approach (NTC01835041). There are currently several drugs that target mitochondrial metabolic intermediates in the cancer sector. The majority of these substances are, however, underutilized in PDAC, which presents an opportunity to examine their efficacy in this illness.

## 10. Antineoplastic Drug Resistance and Metabolism

Glucose, lipid, and amino acid metabolisms are significantly correlated with gemcitabine (chemotherapy drug) resistance [208]. The metabolome of gemcitabine-sensitive or gemcitabine-resistant pancreatic cancer cell lines clearly differs, according to metabolic profiling [209]. Pancreatic cancer cell lines with chemoresistance induced by moderately long-term gemcitabine therapy show increased aerobic glycolysis and decreased ROS levels compared to their parental cells. Enhanced glycolysis keeps ROS levels low, which promotes CSC and EMT phenotypes and increases chemoresistance [210]. Moreover, increased HIF-1α is one factor that contributes to this accelerated glycolysis. Elevated MUC1 expression, a transmembrane protein, stimulates and stabilizes HIF-1α in addition to hypoxia, promoting glycolysis, nonoxidative PPP, and pyrimidine production [109,118]. Gemcitabine resistance develops in pancreatic cancer cells as a result of each of these elements. Due to this mechanism, leflunomide and other pyrimidine biosynthesis inhibitors, such as digoxin or YC1, were able to increase the effectiveness of gemcitabine in animal models. Additionally, MUC1 suppression makes pancreatic cancer cell lines more sensitive to 5-FU [211]. Additionally, the oncogenic KRAS mutation-suppressed pancreatic tumor suppressor F-box and WD repeat domain-containing 7 (FBW7) suppressed glycolysis in pancreatic cancer cells and improved the effectiveness of gemcitabine in xenograft models [212]. LAT2, an oncogenic protein found in pancreatic cancer cells, might stimulate mTOR in a Gln-dependent manner to prevent apoptosis and encourage glycolysis. Both of them result in the phenotype of gemcitabine resistance, although mTOR inhibitor (RAD001) can overcome this resistance. NAMPT is overexpressed in cancer conditions to maintain increased glycolytic activity, also causing gemcitabine resistance. This resistance to sensitivity was abolished by the NAMPT inhibitor (FK866). One of the glucose transporter inhibitors, CG-5, reduces the expression of E2F1 and boosts the effectiveness of gemcitabine in pancreatic cancer cells [213]. In animal models with xenograft tumors, gambogic acid and fructose-1,6-bisphosphatase 1 suppress the ERK signaling pathway and overcome gemcitabine resistance [214,215]. The E2F1-dependent pathway may also be adopted by pancreatic cancer cells treated with gemcitabine, but the exact mechanism is still unclear [213]. By elevating the glycosylation of numerous proteins in various chemoresistant signaling pathways, increased HBP in pancreatic cancer cells also results in gemcitabine resistance [216]. The NF-kB/STAT3 signaling cascade is thought to be activated by low to moderately high ROS levels, sustaining the CSC profile and causing chemoresistance [217]. Low nutritional circumstances, along with gemcitabine, support moderate ROS production that activates the RNA-binding protein HuR. In order to improve NADPH recycling and preserve redox balance, activated HuR quickly upregulates IDH1 [218], and further leads to chemoresistance.

The importance of the milieu in chemoresistance has increased even if there is no evidence linking metabolic interaction in chemoresistance and microenvironment directly. For instance, in murine pancreatic cancer, CAFs might scavenge gemcitabine and promote chemoresistance [219]. According to a different study, highly expressed vitamin D receptors on PSCs limit their ability to sustain malignancies and enhance the delivery and effectiveness of gemcitabine when they bind ligands [220]. Additionally, in pancreatic cancer organoids, metformin improved the effectiveness of oxaliplatin and overcame CAF-induced treatment resistance [221]. Along with CAFs, nab-paclitaxel internalization of TAMs by macropinocytosis may promote macrophage M1 polarization and restore immune recognition in pancreatic cancer [222]. There may be several possibilities to support the anticancer effects of conventional chemotherapy given the vast and crucial metabolic interaction within TME.

Clinical studies have demonstrated that individuals with pancreatic cancer who have high baseline metabolism respond poorly to chemoradiotherapy [223,224]. Radioresistance is significantly aided by increased glycolysis–nucleotide metabolism, which is regulated by upregulated MUC1 in pancreatic cancer [117]. By preventing glucose metabolism, 2-DG can enhance metabolic oxidative stress and lead to the radiosensitization of pancreatic cancer [225]. In mice models with pancreatic cancer xenografts, ketogenic diets characterized by high fat and low carbohydrate intake boosted radiation sensitivity. However, a pertinent phase I clinical trial in individuals with pancreatic cancer that was conducted with little compliance was unsuccessful (NCT01419483) [226].

## 11. Conclusions

The bioenergetic changes that various cancer cell types go through vary, with some becoming more glycolytic and others becoming more oxidative, based in part on the developmental stage of the cell that is enduring neoplastic transformation. There cannot be a single bioenergetic shift that is universal to all cancer cell types, as postulated by Warburg, because of the tissue-specific contextual underpinning of cancer. Because of the resultant variety, it is likely understood why HIF1 may either promote or repress tumor growth, p53 can either promote or inhibit OXPHOS in cancer, and MYC and FOXO can alter mitochondrial biogenesis [96]. The control of nuclear-coded mitochondrial genes and the relationship of bioenergetics to the epigenome [227], the classification of mitochondrial proteins within the mitochondrion [228], the methods by which mitochondrial redox and Ca2+ are regulated and their effect on cytosol and nucleus [227,229], the consequences of mtDNA heteroplasmy and mtDNA–nDNA interactions [230], tissue-based bioenergetics [227,230,231], and the role of cancer cell and stroma interactions [228,232,233,234,235,236,237] all belong to an immature understanding of mitochondria. Targeting altered mitochondrial function or metabolomics in PDAC is supported by the notion that mitochondria are involved in tumor proliferation, metastatic dissemination, ECM and cytoskeleton dynamics, and immune regulation. Major mitochondrial genes including MT-ND3, MT-CO1, IDH2, and TFAM are being reported in various pancreatic cancers, including PDAC (based on cBioportal data), but their actual roles in PDAC progression need further validation. With new information, mitochondria may connect these disparate domains, an important step in the treatment and prevention of cancers. Further, a focus on integrating the diverse functions of mitochondria in cellular activity, how mitochondrial malfunction contributes to illness, and the functions of mitochondria in immunological defense, stem cell formation, and epigenetics will be beneficial in designing novel therapeutics.

## Figures and Tables

**Table 1:** Role of mitochondrial DNA in PDAC progression. (Data source: https://www.cbioportal.org/ accessed on 10 January 2023 [105].)

Mitochondrial	Sample ID	Protein Change	Mutation Type	Variant Type
MT-CO1	ICGC_0054	L367P	Missense Mutation	SNP
	ICGC_0389	E266Nfs	Frame Shift Deletion	DEL
	ICGC_0067	G269E	Missense Mutation	SNP
	ICGC_0016	G457S	Missense Mutation	SNP
	ICGC_0067	G160 *	Nonsense Mutation	SNP
	ICGC_0285	R5H	Missense Mutation	SNP
	ICGC_0046	D445N	Missense Mutation	SNP
	ICGC_0367	D51N	Missense Mutation	SNP
	GARV_0671	V456M	Missense Mutation	SNP
	GARV_0671	V28I	Missense Mutation	SNP
	ICGC_0006	I247T	Missense Mutation	SNP
	ICGC_0102	T181P	Missense Mutation	SNP
	ICGC_0139	S489P	Missense Mutation	SNP
	ICGC_0154	A89T	Missense Mutation	SNP
	ICGC_0188	V155A	Missense Mutation	SNP
	ICGC_0225	A3P	Missense Mutation	SNP
	ICGC_0504	G42S	Missense Mutation	SNP
	ICGC_0112	P500Hfs	Frame Shift Deletion	DEL
	ICGC_0137	A133T	Missense Mutation	SNP
	ICGC_0245	Y371H	Missense Mutation	SNP
	ICGC_0328	M278T	Missense Mutation	SNP
IDH2	TCGA-IB-7651-01	G201D	Missense Mutation	SNP
	TCGA-IB-7651-01	L143M	Missense Mutation	SNP
	TCGA-IB-7651-01	K133R	Missense Mutation	SNP
	TCGA-IB-AAUO-01	R288L	Missense Mutation	SNP
	TCGA-3A-A9IH-01	A239V	Missense Mutation	SNP
	TCGA-IB-7651-01	G201D	Missense Mutation	SNP
	TCGA-IB-7651-01	L143M	Missense Mutation	SNP
	TCGA-IB-7651-01	K133R	Missense Mutation	SNP
MT-ND3	ICGC_0361	V88A	Missense Mutation	SNP
	ICGC_0075	A4T	Missense Mutation	SNP
	ICGC_0343	W113	Missense Mutation	SNP
	ICGC_0008	E38K	Missense Mutation	SNP
	ICGC_0271	A14T	Missense Mutation	SNP
	ICGC_0350	I96T	Missense Mutation	SNP

* The nonsense mutation leading to premature termination of the protein is represented as starred.

**Table 2:** Clinical trials against PDAC using mitochondrial metabolism inhibitors. (Data source: https://clinicaltrials.gov/ [187]. Accessed on 25 January 2023).

Molecular Target	Mitochondrial Inhibitor	Combination with	PDAC Stage	Clinical Trial	NCT Number	Outcome Measures	Status
OXPHOS (Complex1)	Metformin hydrochloride		Resectable	II	NCT02978547	Metformin effect in PDAC proliferation, glucose and insulin metabolism	Unknown
	Metformin	Aspirin, ACE inhibitors, B-blockers	Patients underwent surgical resection or chemotherapy	NA	NCT04245644	DFS; OS	Recruiting
	Metformin		PDAC patients with hyperglycemia	NA	NCT05132244	ORR; PFS; OS	Not yet recruiting
	Metformin	Gemcitabine, Erlotinib	Locally advanced or metastatic	II	NCT01210911	PFS; ORR; toxicity	Completed
	Metformin	Oxaliplatin, Fluorouracil, Leucovorin calcium	Metastatic	II	NCT01666730	ORR and clinical benefit rate based on CT and MRI	Completed
	Metformin	Stereotactic radiosurgery	Borderline-resectable or locally advanced	Early phase I	NCT02153450	Dose-limiting toxicity; PFS using RECIST	Completed
	Metformin	Gemcitabine, Nab-paclitaxel, dietary supplement	Unresectable	I	NCT02336087	Feasibility of Metformin combinations	Completed
	Metformin	Rapamycin	Metastatic, stable disease after FOLFIRINOX or Gemcitabine treatment	I	NCT02048384	Feasibility and safety	Completed
	Metformin	Paclitaxel	Locally advanced or metastatic, after Gemcitabine failure	II	NCT01971034	Time to progression; biochemical response estimation	Completed
OXPHOS (complex IV)	Arsenic trioxide		Locally advanced or metastatic, after Gemcitabine failure	II	NCT00053222	ORR	Completed
PDH and KGDH	Devimistat (CPI-613)	mFOLFIRINOX	Unresectable	II	NCT03699319	OS; MTD; PFS	Completed
	Devimistat (CPI-613)	mFOLFIRINOX	Metastatic	III	NCT03504423	OS; PFS; ORR	Completed
	Devimistat (CPI-613)	Gemcitabine		I	NCT05325281	Maximum tolerated dose; toxicity	Recruiting

DFS, disease-free survival; OS, overall survival; PFS, progression-free survival; ORR, overall response rate; α-KGDH, α-ketoglutarate dehydrogenase; mFOLFIRINOX, modified FOLFIRINOX; OXPHOS, oxidative phosphorylation; PDH, pyruvate dehydrogenase; RECIST, response evaluation criteria in solid tumors; TCA, tricarboxylic acid.
